# Adverse Events Following Immunization Among Children Under Two Years of Age: A Prospective Observational Study From North India

**DOI:** 10.7759/cureus.38356

**Published:** 2023-04-30

**Authors:** Sneha Mittal, CMS Rawat, Amit Gupta, Hariom K Solanki, RK Singh

**Affiliations:** 1 Department of Community Medicine, Soban Singh Jeena Government Institute of Medical Science and Research, Almora, IND; 2 Department of Community Medicine, Veer Chandra Singh Garhwali Government Medical Science and Research Institute, Srinagar, IND; 3 Department of Pharmacology, Soban Singh Jeena Government Institute of Medical Science and Research, Almora, IND; 4 Department of Community Medicine, Government Institute of Medical Sciences, Greater Noida, Greater Noida, IND; 5 Community Medicine, Pandit Deendayal Upadhyaya Medical College, Churu, IND

**Keywords:** india, incidence, prospective studies, vaccines, immunization

## Abstract

Background: Vaccination is one of the most cost-effective child survival health interventions. A single serious adverse event following immunization (AEFI) or a cluster of events may lead to a loss of public confidence in the program and a major setback to immunization coverage. This study was conducted to assess the incidence of AEFIs in children less than two years of age.

Material and Methods: A prospective community-based observational study was conducted in the North Indian state of Uttarakhand from October 2017 to February 2018. A total of 255 children who attended the selected sub-centres for immunization were finally included in the study. Follow-up home visits on the 8th and 30th day of vaccination were carried out to identify any occurrences of AEFIs.

Results: Among 255 children, 212 AEFIs from 152 vaccinated subjects were reported. The majority of the AEFIs were reported in the age group 0-1 years. The incidence of AEFIs was 33.0 per 100 doses of vaccines administered. The most common AEFI reported was fever (101, 47.6%), followed by swelling (53, 25.0%). Among the vaccines, Pentavalent + oral polio vaccine (OPV) (48.8 per 100 doses) was majorly responsible for AEFIs, followed by diphtheria pertussis tetanus (DPT) + measles and rubella (MR) + OPV (46.6 per 100 doses).

Conclusion: Our findings revealed that although the incidence of AEFI reported was high, all of them were minor and no serious AEFIs were identified. The awareness among health professionals and the public regarding the reporting of AEFIs should be continued to increase the safety profile of vaccines.

## Introduction

Vaccination is one of the most cost-effective child survival health interventions [1]. It is one of the best health interventions money can buy and is estimated to save between 3.5 to 5 million lives worldwide each year from diseases like diphtheria, tetanus, pertussis, influenza and measles [2]. Vaccine-preventable diseases are estimated to cause the death of about 0.5 million children in India and another 8.9 million children are at risk of them owing to their either partially immunized or unimmunized status [3]. While immunization is one of the most successful public health interventions, coverage has plateaued over the last decade. Global coverage has dropped from 86% in 2019 to 81% in 2021. An estimated 25 million children under the age of one year did not receive basic vaccines, which is the highest number since 2009 [2].

The Expanded Program on Immunization (EPI) in India was launched in 1978 to eliminate and eradicate the targeted infectious diseases from the country and later in 1985 it was renamed the Universal Immunization Program (UIP) [4]. India’s full immunization coverage has increased to 75.5% in 2020-2021 (NFHS-5) from 62.0% (NFHS-4) [5]. The situation in Uttarakhand has also improved from 57.7% in 2015-16 to 82.0% in 2020-2021 [6]. Vaccines used in national immunization programs are considered safe and effective when used correctly. Vaccines are, however, not risk-free and adverse events will occasionally occur following vaccination [4]. Adverse events following immunization (AEFI) are defined as ‘any untoward medical occurrence which follows immunization and which does not necessarily have a causal relationship with the use of the vaccine.’ The adverse event may be any unfavourable or unintended sign, an abnormal laboratory finding, a symptom, or a disease [7]. However, in the majority of cases, AEFI shows a temporal association between vaccination and adverse events and not a causal relationship between the two. Although most adverse events are minor (e.g. pain, swelling, redness at the injection site, fever), more serious reactions (e.g. seizures, anaphylaxis) can occur, though at a very low frequency [4]. A single serious AEFI or cluster of events may lead to a loss of public confidence in the program and a major setback to immunization coverage. To maintain public trust in the immunization program, AEFI occurrence needs to be minimized. Multiple studies in different parts of India have reported misconceptions about AEFI as a major barrier identified for low immunization coverage [1,8-16].

In India, a limited number of studies on AEFI were found and they were mainly hospital or record-based, and none was from Uttarakhand [17-21]. Hence, the present community-based study aims to study the adverse events following immunization among children less than two years of age receiving vaccines under a routine immunization program.

## Materials and methods

Study design

A prospective community-based observational study was conducted from October 2017 to February 2018. 

Study setting

The study was conducted in the Haldwani block of the Nainital district in Uttarakhand, a state in North India. Nainital is one of six districts in the Kumaon region of Uttarakhand state and has eight blocks, of which, the Haldwani block caters to the largest population of 2.27 lakh. The population of children of age 0-6 years is 26,867 which is 11.8% of the total population. Immunization services to children are provided free of cost in the rural areas of Haldwani by public health facilities which include five primary health centres (PHCs) to which 24 sub-centres are attached [22].

Study population

Children aged less than two years who received vaccines under UIP constituted the study population. The children were accompanied by their mothers; those who gave written informed consent on behalf of their offspring were included in the study.

Sample size

Assuming an average rate of occurrence of AEFIs of any nature to be 50% and a margin of error of 6%, the expected sample size for this study was calculated to be 267. Considering a drop-out rate of 10%, we planned to enrol 296 children.

Sampling technique and study sample

**Figure 1:**
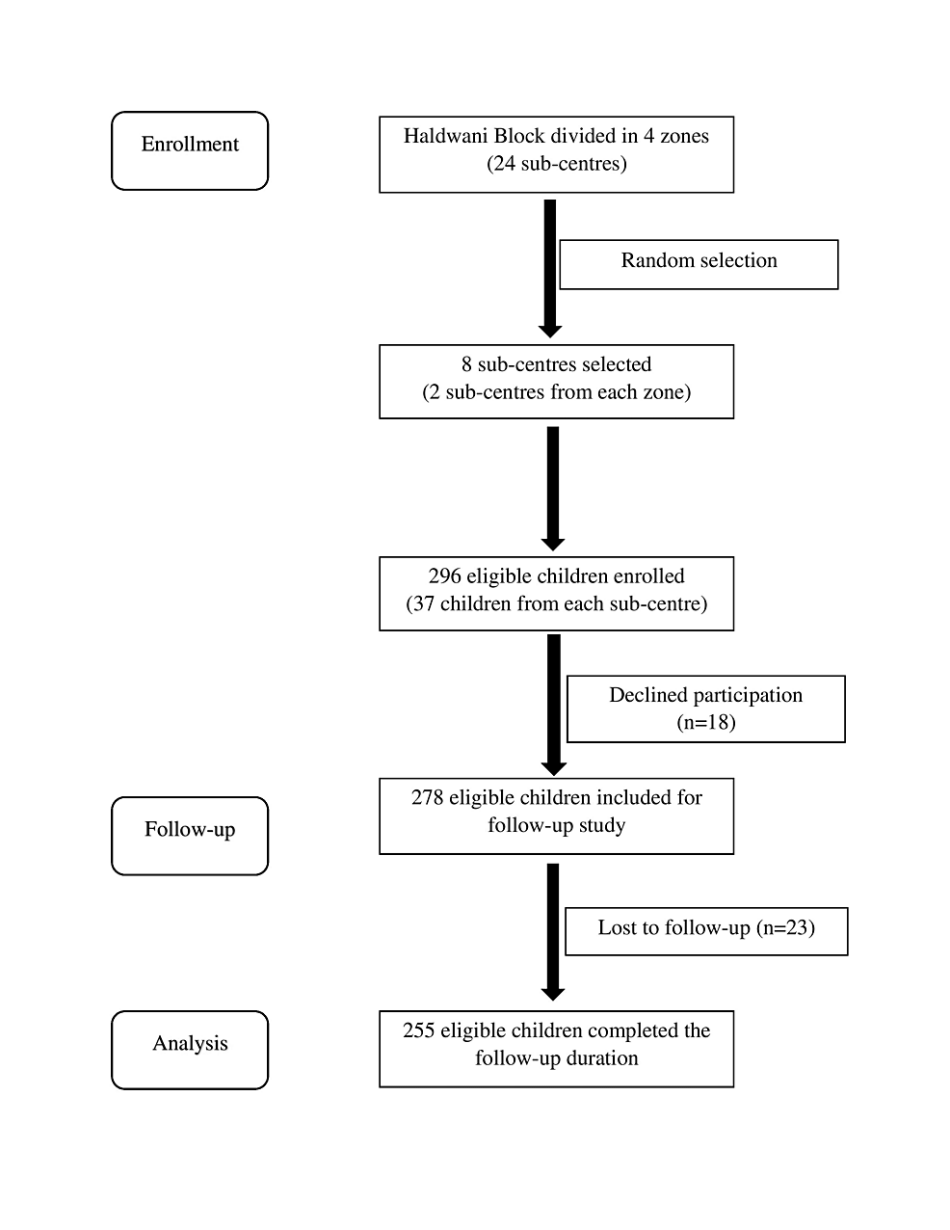
Study flow diagram

Simple random sampling was used for the selection of sub-centres and consecutive sampling for the enrollment of eligible children. The flow diagram of the study sample selection is depicted in Figure 1. 

Data collection

Data was collected using a pretested, semi-structured questionnaire that contained 20 questions regarding the socio-demographic characteristics of the participants, vaccination details, and the AEFI history of the child. The interview of the eligible participants was done by a postgraduate resident of the Community Medicine department which was of around 20-30 minutes duration. All the mothers who came for the vaccination of their child were explained about the study and written informed consent was taken from them. 

A follow-up home visit on the eighth and 30th day of vaccination of the enrolled study subjects was done to identify if any AEFI occurred. If yes, details about AEFI were recorded further using the same questionnaire which was filled out at the session site.

Statistical analysis

Collected data were coded appropriately, entered in a Microsoft Excel spreadsheet, and later cleaned for any possible errors in SPSS Statistics for Windows v.16.0 (IBM Corp., Armonk, NY). Analysis was also carried out using SPSS. Categorical data are presented as a percentage. The descriptive analysis of categorical data is presented as frequencies and percentages. The chi-square test was used to assess the association between two categorical variables and p < 0.05 was considered statistically significant.

Ethical clearance

Ethical clearance was obtained before conducting the study from the Institutional Ethical Committee of Government Medical College of Haldwani, District Nainital, Uttarakhand (Letter number - 338/GMC/IEC/2016/Reg.no.285/IEC/R-19.09.2016 dated 07-10-2016).

## Results

A total of 296 children who received vaccination were screened; from these, the mothers of 18 children refused to participate in the study. Of the enrolled 278 children, 255 of them completed the two follow-up assessments following vaccination with a response rate of 86.1%.

In Table 1, it is shown that out of 255 children included in the study, the majority were in the age group of 0 - 1 year (79.6%), females (51%), Hindu by religion (90.2%), residing in a joint family (64.3%) and had birth weight >2500 grams (52.9%). Almost two-thirds (60.0%) of study subjects were in the upper, upper middle, and middle classes. Mothers of 93.7% of children were literate and 96.1% were housewives.

**Table 1:** Socio-demographic profile of study participants

Variable	Categories	Total (N=255)	Per cent
Age group (years)	0 – 1	203	79.6
	1 – 2	52	20.4
Gender	Male	125	49.0
	Female	130	51.0
Religion	Hindu	230	90.2
	Sikh	18	07.1
	Muslim	7	02.7
Type of family	Nuclear	91	35.7
	Joint	164	64.3
Education of mother	Illiterate	16	06.3
	Literate	239	93.7
Occupation of mother	Housewife	245	96.1
	Employed	10	03.9
Birth weight	< 2500gm	94	36.9
	> 2500gm	135	52.9
	Not known	26	10.2
Socioeconomic status	Upper	20	07.8
	Upper middle	64	25.1
	Middle	69	27.1
	Lower middle	76	29.8
	Lower	26	10.2

Of the 642 vaccine doses administered to the study population, 212 AEFI were reported from 152 vaccinated subjects. The incidence of AEFI in our study was 33.0 per 100 doses of vaccines administered. Out of 152 children with AEFI, the majority (77.6%) were aged less than one year and the gender-wise distribution was almost similar with male to female ratio of 1.03. There was no significant association of AEFI with age and gender (p > 0.05) as shown in Table 2.

**Table 2:** Distribution of AEFI in children according to age and gender

Variable	Categories	Total (N=255)	Children with AEFI n (%)	P-value
Age group (years)	0 – 1	203	118 (58.12)	0.427
	1 – 2	52	34 (65.38)	
Gender	Male	125	77 (61.60)	0.611
	Female	130	75 (57.69)	

Table 3 shows that AEFI incidence following administration of Pentavalent + oral poliovirus vaccines (OPV) at the age of 10 weeks was highest (48.8 per 100 doses) followed by the vaccines for diphtheria pertussis tetanus (DPT) + measles and rubella (MR) + OPV (46.6 per 100 doses) at 16 to 24 months of age. Following Pentavalent+inactivated polio vaccine (IPV) + OPV administration, the incidence was 43.3 per 100 doses when administered at the age of six weeks whereas it was 28.2 per 100 doses at the age of 14 weeks. No AEFI was reported after bacillus Calmette‑Guerin vaccine (BCG) + Hepatitis B + OPV administration at less than one week of age. The least incidence (07.7 per 100 doses) was with measles/MR at nine months to one year of age. Overall AEFI incidence reported was 33.0 per 100 doses.

**Table 3:** Distribution of AEFI rate per 100 doses according to vaccines administered BCG: Bacillus Calmette-Guerin vaccine; hepatitis B: hepatitis B vaccine; OPV: oral polio vaccine; IPV: inactivated polio vaccine; Pentavalent: pentavalent vaccine; measles: measles vaccine; MR: measles and rubella vaccine; DPT: diphtheria pertussis tetanus vaccine

Age	Vaccines administered	Doses of vaccines (b)	No. of AEFIs (a)	AEFI incidence per 100 doses [(a/b)*100]
<1 week	BCG+Hepatitis-B+OPV	87	0	0.0
6 weeks	Pentavalent+IPV+OPV	150	65	43.3
10 weeks	Pentavalent+OPV	90	44	48.8
14 weeks	Pentavalent+IPV+OPV	156	44	28.2
9 months	Measles / MR	39	03	07.7
16-24 months	DPT+MR+OPV	120	56	46.6
Total		642	212	33.0

Table 4 shows that out of a total of 212 AEFIs, fever was the most common AEFI (101; 47.6%) with most of the cases of it after administration of Pentavalent+IPV+OPV vaccines (45; 44.6%), followed by swelling at the injection site (53; 25.0%) with most cases of it after administration of Pentavalent+IPV+OPV vaccine (38; 71.7%), and the least common was nodule (0.5%).

**Table 4:** Frequency distribution of various AEFI according to suspected vaccines BCG: Bacillus Calmette-Guerin vaccine; hepatitis B: hepatitis B vaccine; OPV: oral polio vaccine; IPV: inactivated polio vaccine; Pentavalent: pentavalent vaccine; measles: measles vaccine; MR: measles and rubella vaccine; DPT: diphtheria pertussis tetanus vaccine

Vaccines	Fever		Excessive Cry		Swelling		Pain		Redness		Nodule		Total	
	n	%	n	%	n	%	n	%	n	%	n	%	n	%
BCG + Hepatitis B + OPV	0	0.0	0	0.0	0	0.0	0	0.0	0	0.0	0	0.0	0	0.0
Pentavalent + IPV + OPV	45	44.6	25	73.5	38	71.7	0	0.0	1	50.0	0	0.0	109	51.4
Pentavalent + OPV	25	24.8	9	26.5	9	17.0	0	0.0	1	50.0	0	0.0	44	20.8
Measles / MR	2	2.0	0	0.0	1	1.9	0	0.0	0	0.0	0	0.0	3	1.4
DPT + MR + OPV	29	28.7	0	0.0	5	9.4	21	100.0	0	0.0	1	100.0	56	26.4
Total	101	47.6	34	16.0	53	25.0	21	9.9	2	0.9	1	0.5	212	100.0

It was observed that all the AEFI were reported in the interval between the day of vaccine administration and the first follow-up visit (eighth day), and none was reported between the first and second follow-up visits. The study found that 98% of children recovered at the time of the first follow-up visit and all of them recovered at the second follow-up visit.

## Discussion

Immunization is an important public health intervention for improving child health by reducing morbidity and mortality due to vaccine-preventable diseases. Although vaccines are safe, they are not completely risk-free, and adverse events may occasionally follow. Irrespective of the nature, minor or major, adverse events cause anxiety and lead to loss of public confidence in the universal immunization program. The present work was done to study the adverse events following immunization among children less than two years of age.

In our study, all adverse events appeared within a few days of vaccination and 98% of study subjects recovered on the day of the first follow-up visit on the eighth day) whereas all recovered at the time of the second follow-up visit (30th day) which is in agreement with Sebastian et al. study [20].

In our study, the majority of AEFI were among children in the age group of less than 1 year (77.6% AEFI). These results were expected, as maximum doses of vaccines according to National Immunization Schedule are given in this age group. The results are comparable to a study by Aherkar et al. and Badur et al., where the most common age group was less than nine months and one year respectively [18,21]. The studies by Patel et al., Cunha et al., and Dey et al., also reported the most common age group for AEFI occurrence to be less than one year [23-25].

In our study, of the total AEFI, the most common was fever (47.6%), followed by swelling at the injection site (25.0 %) as in previously published studies [18,20,21].

Overall Pentavalent + OPV (48.8 per 100 doses) was majorly responsible for AEFI followed by DPT + MR + OPV (46.6 per 100 doses) which is consistent with the findings of other studies [20,21]. In the present study, no AEFI was reported after BCG + hepatitis B + OPV while in a study by Badur et al., 35.5% AEFI were reported; this significant difference in the AEFI rate as compared to our study could be on account of the other study having a large sample size (20,414 children) with the study sample including about 70% children receiving BCG + hepatitis B + OPV vaccine [21].

The incidence of AEFI in our study was 33.3 per 100 doses of all vaccines which was significantly high when compared to that reported by Aherkar et al. (8.61 per 100 doses), Sebastian et al. (13.7%) and Badur et al. (13.6/10,000 doses) [18,20,21]. In a study by Patel et al., the AEFI rate was 8.3 per 100,000 doses [23] and Danova et al. reported an AEFI rate of 209 per 100000 doses [26]. This variation in rates of reported AEFI in different studies could be explained by the type of surveillance, the type of vaccines under study, variable case definitions, reporting criteria, variable compliance with reporting, and sample size of the study population. Although the incidence of AEFI reported was high, all of them were minor and no serious AEFI was identified in our study, while others, like Joshi et al., reported 0.7% AEFIs to be serious [27] and Arora et al. reported 365 hospitalizations and 17 deaths during four-week follow-up [28].

Limitations

The study was conducted in one block of the district and included children immunized at government health centres; it limits the generalization of the study findings. There was the possibility of recall bias as the identification of AEFI was based on history taken from mothers which could lead to under or over-reporting of AEFI. It was not possible to identify which vaccine was responsible for many of the AEFIs since multiple vaccines were administered to the children at each of the immunization visits. Another limitation of the study was a small sample size due to declined participation and loss to follow-up which may contribute to potential selection bias. 

## Conclusions

The incidence of AEFI was high (33.3 per 100 doses) in the present study but no serious adverse events attributable to vaccines were reported. The study revealed that the vaccine were safe and well-tolerated as the majority of the children recovered spontaneously without any formal consultation. The awareness among health professionals and the public regarding reporting of AEFI should be continued to increase the safety profile of vaccines, and to increase public trust in immunization programs.
